# The genetic basis for inactivation of Wnt pathway in human osteosarcoma

**DOI:** 10.1186/1471-2407-14-450

**Published:** 2014-06-18

**Authors:** Xiaoling Du, Jilong Yang, Da Yang, Wei Tian, Ze Zhu

**Affiliations:** 1 Department of Diagnostics, Tianjin Medical University, Tianjin 300060, China; 2 Department of Bone and Soft Tissue Tumors, Tianjin Medical University Cancer Institute & Hospital, National Clinical Research Center for Cancer, Tianjin 30060, China; 3 Department of Pathology, The University of Texas MD Anderson Cancer Center, Houston, TX 77030, USA; 4 Department of Medical Microbiology, Tianjin Medical University, Tianjin 300060, China

**Keywords:** Osteosarcoma, Wnt signal pathway, Genetic aberration, Microarray-based comparative genomic hybridization

## Abstract

**Background:**

Osteosarcoma is a highly genetically unstable tumor with poor prognosis. We performed microarray-based comparative genomic hybridization (aCGH), transcriptome sequencing (RNA-seq), and pathway analysis to gain a systemic view of the pathway alterations of osteosarcoma.

**Methods:**

aCGH experiments were carried out on 10 fresh osteosarcoma samples. The output data (Gene Expression Omnibus Series accession number GSE19180) were pooled with published aCGH raw data (GSE9654) to determine recurrent copy number changes. These were analyzed using Kyoto Encyclopedia of Genes and Genomes (KEGG) pathway analysis to identify altered pathways in osteosarcoma. Transcriptome sequencing of six osteosarcomas was performed to detect the expression profile of Wnt signaling pathway genes. Protein expression of WNT1, β-catenin, c-myc, and cyclin D1 in the Wnt pathway was detected by immunohistochemistry (IHC) in an independent group of 46 osteosarcoma samples.

**Results:**

KEGG pathway analysis identified frequent deletions of *Wnt* and other Wnt signaling pathway genes. At the mRNA level, transcriptome sequencing found reduced levels of mRNA expression of Wnt signaling pathway transcripts. While WNT1 protein expression was detected by IHC in 69.6% (32/46) of the osteosarcomas, no β-catenin protein was detected in the nucleus. β-catenin protein expression was, however, detected in the membrane and cytoplasm of 69.6% (32/46) of the osteosarcomas. c-myc protein expression was detected in only 47.8% (22/46) and cyclin D1 protein expression in 52.2% (24/46) of osteosarcoma samples. Kaplan-Meier survival analysis showed that WNT1-negative patients had a trend towards longer disease free survival than WNT1-positive patients. Interestingly, in WNT1-negative patients, those who were also cyclin D1-negative had significantly longer disease free survival than cyclin D1-positive patients. However, there was no significant association between any of the investigated proteins and overall survival of human osteosarcoma patients.

**Conclusions:**

Frequent deletions of *Wnt* and other Wnt signaling pathway genes suggest that the Wnt signaling pathway is genetically inactivated in human osteosarcoma.

## Background

Osteosarcoma is a malignant bone tumor, often associated with copy number alterations, that most commonly arises in the metaphyseal ends of long bones [1-3]. The survival of patients with osteosarcoma has not improved significantly in recent years and the prognosis of patients with metastatic osteosarcoma is especially poor [1]. Identification of prognosis markers and key genetic and molecular events for osteosarcoma is critical for development of effective therapeutics [2,3].

Fortunately, the discovery of signal transduction pathways and their importance in a variety of cancers has led to the development of many new targeted agents. With regard to osteosarcoma, preclinical investigations targeting the rapamycin (mTOR) pathway, as well as the VEGF pathway showed promising results [2,4]. The Wnt pathway is clearly important in many forms of human cancer, particularly in epithelial cancer types where gain- or loss-of-function events appear to contribute to both inherited cancer risk and somatic carcinogenesis [5]. In human osteosarcoma, most previous studies have suggested that active Wnt signaling contributes to osteosarcoma development, evidenced by cytoplasmic and/or membranous β-catenin staining or detection of Wnt pathway components [6,7]. However, Cai and colleagues recently reported that the Wnt pathway is inactivated in osteosarcomas [8]. These contradictory findings provoke debate and stimulate further research into the role of Wnt signaling in osteosarcoma [5].

In this study, we sought to gain a comprehensive understanding of the key driving pathways for osteosarcoma by performing pathway analysis of recurring gene copy number aberrations using array comparative genomic hybridization (aCGH) analysis. Transcriptome sequencing (RNA-seq) and immunohistochemistry (IHC) were used to explore the expression level of key signaling pathways affected by these genetic aberrations. The most surprising and intriguing finding was the deletion of Wnt pathway genes, suggesting the genetic inactivation of the Wnt signaling pathway, in human osteosarcoma.

## Methods

### Osteosarcoma tissues and clinical information

Ten frozen osteosarcoma biopsy samples, all with at least 90% tumor content, were obtained from the Tissue Bank of the Tianjin Medical University Cancer Institute & Hospital (TMUCIH) for aCGH and RNA-Seq analysis as described below. Tumor samples were snap frozen in liquid nitrogen. In addition, an independent group of 46 primary osteosarcoma cases with formalin-fixed and paraffin-embedded (FFPE) tissues and clinicopathologic data were collected. All of the tissues and information collection took place at Tianjin Medical University Cancer Institute & Hospital (TMUCIH) with Institutional Review Board (IRB) approved protocols and the patients’ consent. The clinical and pathological parameters included age, gender, locations, Enneking staging, and follow-up data (Table 1). All neoadjuvant and adjuvant chemotherapy had been administered according to the Rosen T10 regimen [9,10]. Disease-free and overall survival time ranged from 0 to 94 months, with medians of 9 and 13 months, respectively.

**Table 1:** The clinical and pathological features in 46 human conventional osteosarcomas

		**N**	**Disease free survival**				**Overall survival**			
			**P**	**HR**	**95.0% CI**		**P**	**HR**	**95.0% CI**	
					**L**	**U**			**L**	**U**
Sex	Male	29	0.86				0.07*			
	Female	29	0.86	1.11	0.36	3.44	0.08	3.49	0.86	14.28
Age group	<=15 y	14	0.76				0.79			
	15-20 y	23	0.94	1.10E + 04	0	1.40E + 116	0.5	1.82	0.32	10.42
	21-30 y	11	0.94	3.70E + 04	0	4.40E + 116	0.72	0.73	0.13	4.05
	31-40 y	1	0.93	5.30E + 04	0	6.40E + 116	0.97	0	0	1.90E + 282
	>40 y	9	1	1.02	0	3.90E + 225	0.99	0	0	
Tumor location	limbs	51	0.28				0.97			
	Others	7	0.48	23.34	0.004	1.60E + 05	0.97	0.97	0.12	7.72
Enneking stage	I	4	0.96				0.43			
	IIA	19	0.78	0.71	0.06	7.88	0.17	0.1	0.004	2.74
	IIB	2	0.631	0.59	0.07	5.01	0.1	0.09	0.01	1.61
	III	1	0.897	0.83	0.05	13.39	0.99	0	0	
Neoadjuvant chemotherapy	No	10	0.51				1.5E-4*			
	Yes	30	0.51	1.51	0.44	5.15	0.003	11.85	2.36	59.45
Adjuvant chemotherapy	No	3	0.21				0.41			
	Yes	19	0.43	0.04	0	129.34	0.61	0.04	0	1.20E + 04
Recurrence	No	50					0.49			
	Yes	8					0.49	2.05	0.26	16.26
Metastasis	No	38					0.045*			
	Yes	11					0.436	0.01	0	2.40E + 03

*Significantly different; L: lower; U: upper.

### Array comparative genomic hybridization and pathway enrichment analysis

The aCGH data analysis was performed as previously described [2,3]. aCGH data in this publication have been deposited in NCBI’s Gene Expression Omnibus (GEO) and are accessible through GEO Series accession number GSE19180. In addition, we obtained the raw aCGH data of another 10 osteosarcoma biopsy samples from the GEO database (GSE9654) [11] and pooled the two datasets for analysis. Briefly, the median-normalized log-2 ratio data were first subjected to a circular binary segmentation (CBS) algorithm to reduce the effect of noise [12]. Then, the CGHcall algorithm was used to call segments of DNA sequences as amplified or deleted in each sample [13]. A permutation analysis was further applied to call recurrent copy number aberration in osteosarcoma [2]. As a result of this procedure, each target was given a label of “normal”, “deletion” or “amplification” as previously described [2,3,13]. A bacterial artificial chromosome (BAC) clone–based CGH array dataset from 36 cases of osteosarcoma was also analyzed to confirm the overall recurrent gene copy alteration patterns [2,3,14].

Pathway enrichment analysis was performed separately on the recurrent amplified and deleted gene lists from gene sets GSE19180 and GSE9654 as previously reported [2,3]. Here, we used the gene annotations data in the Kyoto Encyclopedia of Genes and Genomes (KEGG). Pathways with more annotations in our gene list than expected by random (hypergeometric model, P < 0.05) were considered to be significantly enriched with amplified or deleted genes [2,3].

### RNA-seq analysis

Frozen tumors were crushed, then total RNA was isolated using TRIzol reagent (Invitrogen, Grand Island, NY). RNA was quantified by Qubit (Invitrogen, Grand Island, NY) and Nanodrop ND1000 (ThermoFisher Scientific, Waltham, MA) before quality assessment with the Agilent 2100 Bioanalyzer. Six of the 10 samples with high quality RNA were used for RNA library construction, followed by emulsion PCR and whole transcriptome 90 bp paired-end sequencing on Illumina HiSeq™ 2000 instruments at the Beijing Genomics Institute (BGI, Shenzhen, China). Each sequencing run produced approximately 50 million paired end reads.

Whole transcriptome sequencing reads were aligned against the GRCh37 human reference genome using Tophat version 2.0.4 [15]. The number of overlapping reads was calculated for all exons and then for all genes annotated in Ensembl 67. Gene expression values were normalized across samples using median-of-ratios normalization [16]. Briefly, an expression ratio between two samples for every gene (or exon) with > 500 reads is calculated, and the median of those ratios is determined. All gene expression values are then multiplied by the median-of-ratios.

### Immunohistochemical analysis of WNT1, β-catenin, c-myc, and cyclin D1 expression

The 46 FFPE tissues were sectioned at 4 μm and mounted onto charged glass slides (ProbeOn Plus, Fisher Scientific, Pittsburgh, PA, USA) for immunohistochemical staining as described previously [2]. Briefly, tissues were deparaffinized with xylene and ethanol, endogenous peroxidase activity was blocked with 0.3% H_2_O_2_ (Fisher Scientific, Fair Lawn, NJ, USA). Tissues were blocked for 30 min with normal serum (Vector Laboratories, Burlingame, CA, USA) and then incubated overnight at 4°C with appropriate antibodies. Antibodies for WNT1, β-catenin, c-myc, and cyclin D1 (Abcam, Cambridge, UK) were used at a dilution of 1:200, 1:100, 1:100, and 1:100, respectively. The same concentrations of non-immune rabbit serum were used as negative controls. Signal was detected using biotinylated anti-rabbit antibodies, followed by avidin, biotinylated enzyme and colorimetric detection using 3,3′-diaminobenzidine tetrahydrachloride (DAB) (DAKO Corporation, Carpinteria, CA, USA). Samples were then counterstained with Mayer’s hematoxylin (Polyscientific, Bay Shore, NY, USA).

Two pathologists, blinded to clinical information, evaluated and scored the immunohistochemical staining for WNT1, β-catenin, c-myc, and cyclin D1 in osteosarcoma tissues based on the overall intensity of membranous, cytoplasmic, and nuclear staining within the tumor cells and the percentage of cells stained [8,17-20]. WNT1 and β-catenin staining were scored as described in [8,17]. Specifically, intensity of staining was graded as follows: lost (score 0); weak (“+”, score 1); moderate (“++”, score 2); and strong (“+++”, score 3). Extent of staining was evaluated as the percentage of positive cells per 100 or more cells (at least 100 cells in 10 high-power fields) in each evaluated compartment and was graded into five classes as follows: < 5% (score 0); 6% to 25% (score 1); 26% to 50% (score 2); 51% to 75% (score 3); > 75% (score 4). A final staining score was calculated by adding intensity score and extent score for each compartment, and the expression was categorized into two classes based on the final score: positive (final score 2–7) and negative (final score 0–1) expression. For β-catenin staining evaluation, any intensity and extent of staining found in the nucleus was considered positive. The staining of c-myc and cyclin D1 was assessed on the basis of published data: negative (< 10% of the cells), and positive (> 10% of the cells) [18-20].

### Statistics

Student’s t-test, ANOVA, Chi-square, Fisher’s exact test, Kaplan-Meier, and Mantel-Cox survival analysis were performed using SPSS software 16.0 version when necessary. A P value of less than 0.05 was considered statistically significant in multivariate analysis.

## Results

### Several signaling pathways are genetically altered in osteosarcoma

**Figure 1:**
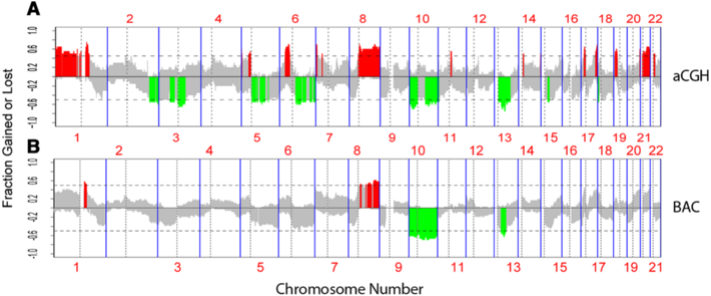
**Genetic aberrations in human osteosarcoma samples.** The x-axis numbered with 1–22 denotes chromosome numbers. The y-axis denotes log-ratio for every aCGH probe (scatter plot). The y-axis shows recurrence of gains (positive axis) and losses (negative axis) for each measured sequence aligned evenly in chromosomal order on the x-axis. Recurrence rates that exceed the threshold are color-coded to emphasize the locations of significantly recurrent aberrations. Red denotes significantly recurrent amplifications and green denotes significantly recurrent deletions. Gray represents nonsignificant recurrence of aberrations. **A**. Genetic aberrations of 20 osteosarcomas (GSE19180 and GSE9654). **B**. Genetic aberrations of 36 Norwegian osteosarcoma samples.

As presented in our previous reports, high-density genome-wide aCGH profiling (GSE19180) of 10 osteosarcoma tissues identified several major regions with significant genetic alterations. This pattern of copy number alterations was strikingly similar to that from an independent aCGH dataset (GSE9654) of osteosarcoma samples obtained from Canadian patients [2,3,11]. It was therefore reasonable to pool the two aCGH datasets (GSE19180 and GSE 9654) for further pathway analysis. Analysis of the combined dataset led to the identification of the amplification of 2,519 genes and deletion of 1,276 genes among the 20 osteosarcoma samples (Figure 1A). We also analyzed a BAC clone-based, aCGH dataset from 36 Norwegian osteosarcoma samples (Figure 1B) [14]. The analysis of this third dataset showed similar results to the first 20 aCGH osteosarcomas, suggesting consistent genetic alterations underlying the pathogenesis of osteosarcoma.

The GSE19180 and GSE9654 datasets were measured on Agilent Human Genome CGH Microarrays, while the third aCGH dataset was BAC clone-based. Therefore the KEGG pathway enrichment analysis was only performed on dataset GSE19180 and GSE9654. In the KEGG pathway enrichment analysis, significant genetic amplifications were identified in the component genes of 20 signaling pathways including the VEGF, mTOR, CAMs, and adherens junction signaling pathways. Significant genetic deletions were identified in the component genes of 11 signaling pathways including the Wnt and Hedgehog signaling pathways (Tables 2 and 3). In our previous studies, the integrated methods of aCGH, fluorescent in situ hybridization (FISH), and IHC has led to validation of the genetic amplification of VEGF pathway genes [2].

**Table 2:** Genetic amplifications of key pathway genes in osteosarcoma

**KEEG pathways**	**P-value**	**Gene name cohort**
VEGF signaling pathway	0.000611811	*MAPK14, AKT1, PLA2G2D, PLA2G2E, NFATC4, PIK3CD, PLA2G2A, PLA2G5, MAPK1, MAPK13, PTK2, RAC1, RAC3, PLA2G2F, VEGFA, CASP9, PIK3R3, SPHK1, SH2D2A, CDC42P2, CDC42*
mTOR signaling pathway	0.024894273	*AKT1, MTOR, RICTOR, PIK3CD, PRKAA1, PRKAA2, MAPK1, RPTOR, RPS6KA1, VEGFA, PIK3R3, ULK2*
Tight junction	0.044332801	*INADL, EXOC3, CSNK2B, CLDN19, AKT1, CLDN14, CLDN17, LLGL2, LLGL1, MYH6, MYH7, F11R, ASH1L, PRKCZ, CGN, JAM2, RAB3B, RAB13, ACTB, ACTG1, CLDN5, CLDN8, CRB3, TJAP1, CDC42P2, CDC42*
Synthesis and degradation of ketone bodies	0.019871373	*HMGCL, HMGCS1, HMGCS2, OXCT1*
C21-Steroid hormone metabolism	0.04221587	*CYP11B1, CYP11B2, HSD3B1, HSD3B2*
Peptidoglycan biosynthesis	0.017208179	*PGLYRP2, PGLYRP3, PGLYRP4*
Ether lipid metabolism	0.006547877	*AGPAT1, PLA2G2D, PLA2G2E, PAFAH2, ENPP2, PLA2G2A, PLA2G5, AGPAT3, PLA2G2F, PPAP2B*
Arachidonic acid metabolism	0.02227667	*CYP2J2, CYP4A11, GPX6, PLA2G2D, GPX5, GPX7, PLA2G2E, CYP4F3, PLA2G2A, PLA2G5, PLA2G2F, CYP4F2, CBR3*
Alpha-Linolenic acid metabolism	0.003362937	*PLA2G2D, PLA2G2E, ACOX1, PLA2G2A, PLA2G5, PLA2G2F, FADS2*
Nitrogen metabolism	0.007842327	*CTH, CA14, CA1, CA2, CA3, CA6, CA8, ASRGL1*
Glycan structures-biosynthesis 2	0.025972282	*PIGK, B3GALT5, ST6GALNAC3, PIGP, PIGV, ST3GAL1, ST3GAL3, ST6GALNAC5, B4GALT3, B4GALT2, B3GALT4, GPAA1, PIGM, PIGL*
Biosynthesis of unsaturated fatty acids	0.021314211	*ACOT7, FASN, ACOT11, FADS1, ACOX1, FADS2, TECR*
Antigen processing and presentation	0.028228218	*CTSS, HLA-DMA, HLA-DMB, HLA-DOA, HLA-DOB, HLA-DPA1, HLA-DQA2, HLA-DRB5, HLA-F, HSPA1B, HSPA1A, HSPA1L, HSP90AB1, NFYA, NFYC, RFX5, TAP1, TAP2, CALR*
Fc epsilon RI signaling pathway	0.000481297	*MAPK14, AKT1, FCER1A, FCER1G, PLA2G2D, GRB2, PLA2G2E, LYN, PIK3CD, PLA2G2A, PLA2G5, MAPK1, MAPK13, MAP2K3, MAP2K7, RAC1, RAC3, PLA2G2F, TNF, VAV1, PIK3R3*
Insulin signaling pathway	0.047775005	*FLOT1, CRKL, AKT1, FASN, EXOC7, MTOR, GRB2, INSR, PFKL, PIK3CD, PKLR, PRKAA1, PRKAA2, PRKAB2, PRKACA, PRKACB, PRKCZ, MAPK1, RPTOR, PTPRF, SHC1, SREBF1, PIK3R3, MKNK1, TRIP10*
GnRH signaling pathway	0.04873803	*ADCY8, MAPK14, ADCY4, PLA2G2D, GRB2, PLA2G2E, ITPR3, JUN, PLA2G2A, PLA2G5, PRKACA, PRKACB, MAPK1, MAPK7, MAPK13, MAP2K3, MAP2K7, PLA2G2F, CDC42P2, CDC42*
Alzheimer’s disease	0.020850782	*NCSTN, BACE2, APP, APH1A, C1QA, TNF, C1QB, C1QC*
Asthma	0.003038215	*FCER1A, FCER1G, HLA-DMA, HLA-DMB, HLA-DOA, HLA-DOB, HLA-DPA1, HLA-DQA2, HLA-DRB5, TNF*
Systemic lupus erythematosus	2.49E-06	*FCGR1A, FCGR2A, FCGR2B, FCGR3A, HIST1H2AB, HIST1H2AE, HIST1H2BD, H3F3B, H3F3A, HLA-DMA, HLA-DMB, HLA-DOA, HLA-DOB, HLA-DPA1, HLA-DQA2, HLA-DRB5, HIST2H2AB, C1QA, TNF, C1QB, C1QC, C2, C3, C6, C7, C8A, C8B, C9, HIST1H2AG, SMCHD1, HIST1H2AI, HIST1H2AK, HIST1H2AL, HIST1H2AM, HIST1H2AJ, HIST1H2AC, HIST2H2AC, HIST1H2BC, HIST1H2BE, HIST1H2BF, HIST1H2BG, HIST1H2BI, HIST1H2BO, HIST2H2BE, HIST1H3A, HIST1H3B, HIST1H3C, HIST1H3D, HIST1H3E, HIST1H3F, HIST1H3G, HIST1H3H, HIST1H3I, HIST1H3J, HIST4H4, HIST1H4A, HIST1H4B, HIST1H4C, HIST1H4D, HIST1H4E, HIST1H4F, HIST1H4H, HIST1H4I, HIST1H4J, HIST1H4K, HIST1H4L, HIST2H4A, HIST2H4B, HIST1H4G, HIST1H2AH, HIST1H2BK, HIST1H2BJ*
Allograft rejection	0.046891168	*HLA-DMA, HLA-DMB, HLA-DOA, HLA-DOB, HLA-DPA1, HLA-DQA2, HLA-DRB5, HLA-F, TNF*

**Table 3:** Genetic deletions of key pathway genes in osteosarcoma

**KEEG pathways**	**P value**	**Gene name cohort**
Wnt signaling pathway	0.01039	*FRAT1, CSNK1A1L, CTBP2, PRICKLE2, FRAT2, GSK3B, PLCB2, SFRP5, MAP3K7, TCF7L2, WNT5A, WNT6, WNT8B, FZD5, WNT10A, FZD7, FZD8, BTRC*
Hedgehog signaling pathway	0.012811	*CSNK1A1L, STK36, GSK3B, SUFU, WNT5A, WNT6, WNT8B, WNT10A, BTRC*
Adherens junction	0.014329	*SORBS1, WASF3, FER, FYN, PVRL3, MLLT4, PARD3, MAP3K7, TCF7L2, VCL, WASF1*
Metabolism of xenobiotics by cytochrome P450	0.006437	*AKR1C4, GSTO2, CYP2C19, CYP2C8, CYP2C9, CYP2C18, AKR1C1, AKR1C2, UGT1A8, AKR1C3, GSTO1*
PPAR signaling pathway	0.039963	*SORBS1, CYP27A1, FABP7, ACSL3, ACADL, ME1, ACSL5, SCD, ACOX2*
Non-homologous end-joining	0.001607	*DNTT, POLL, DCLRE1C, XRCC5, NHEJ1*
Phosphatidylinositol signaling system	0.040426	*DGKH, INPP5D, PLCE1, CALML5, PIK3R1, GNG7, PIP4K2A, PLCB2, PTEN, PLCD4, DGKD*
ECM-receptor interaction	0.032505	*COL6A3, FNDC3A, SV2C, FN1, ITGA1, ITGB1, LAMA4, THBS1, THBS2, ITGA8, CD47*
T cell receptor signaling pathway	0.020903	*RASGRP1, CHUK, MAP3K8, CTLA4, FYN, ICOS, NFKB2, PIK3R1, PRKCQ, PAK6, CBLB, CD28*
Melanogenesis	0.007761	*ADCY5, CREB1, GSK3B, MITF, CALML5, PLCB2, TCF7L2, WNT5A, WNT6, WNT8B, FZD5, WNT10A, FZD7, FZD8*
Basal cell carcinoma	0.00088	*STK36, GSK3B, SUFU, TCF7L2, WNT5A, WNT6, WNT8B, FZD5, WNT10A, FZD7, FZD8*

### Wnt pathway genes are deleted in human osteosarcomas

**Figure 2:**
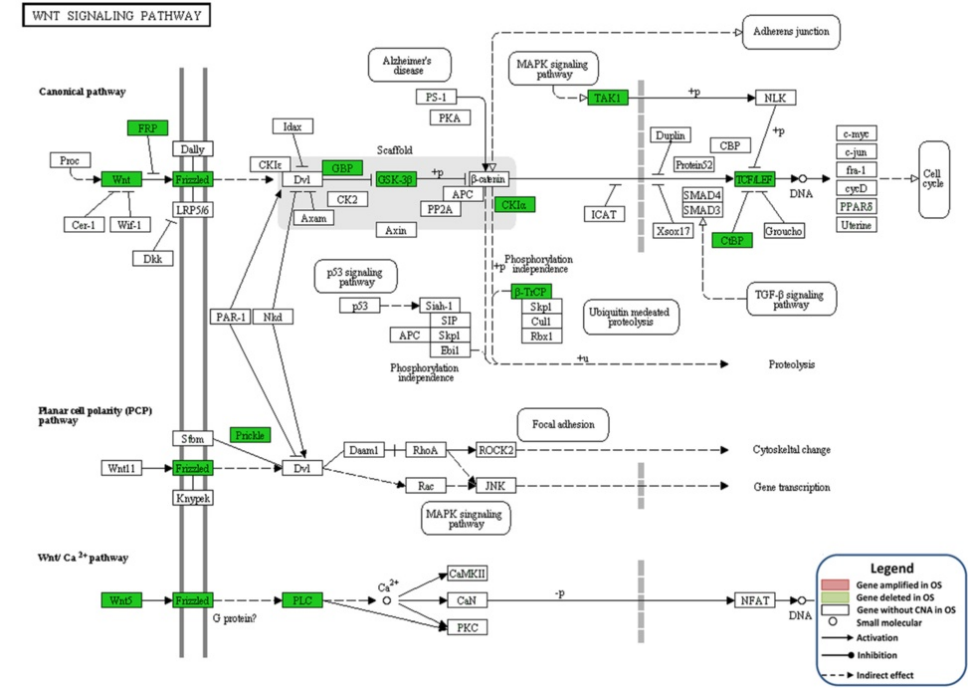
**Visualization of the location of altered genes in the Wnt pathway.** Pink indicates genes with significantly recurrent amplification, and green denotes genes with significantly recurrent deletion. White indicates genes with no significant aberrations.

In contrast to the amplification of VEGF pathway genes, we detected significant over-representation of deleted genes in 11 pathways. Among these pathways, the Wnt signaling pathway was most highly affected (Table 3). This is the first description of this genetic aberration in human osteosarcoma. As controversy exists in the field as to whether the Wnt signaling pathway is inactivated or not in osteosarcoma [8,21], we further investigated copy number alterations of individual genes in the Wnt signaling pathway. In the canonical Wnt signaling pathway, the following genes were significantly deleted across the osteosarcoma dataset: *WNT, FRP, Frizzled, GBP, GSK-3β, TCF/LET, TAK1, CK1, CtBP,* and *B-TrCP* (Figure 2). Specifically, the *WNT1* gene was deleted in 10 cases of the 20 human osteosarcomas with a deletion frequency of 50%.

### Reduced transcript and protein expression of Wnt signaling pathway components suggests the Wnt signaling pathway is inactivated in human osteosarcomas

**Figure 3:**
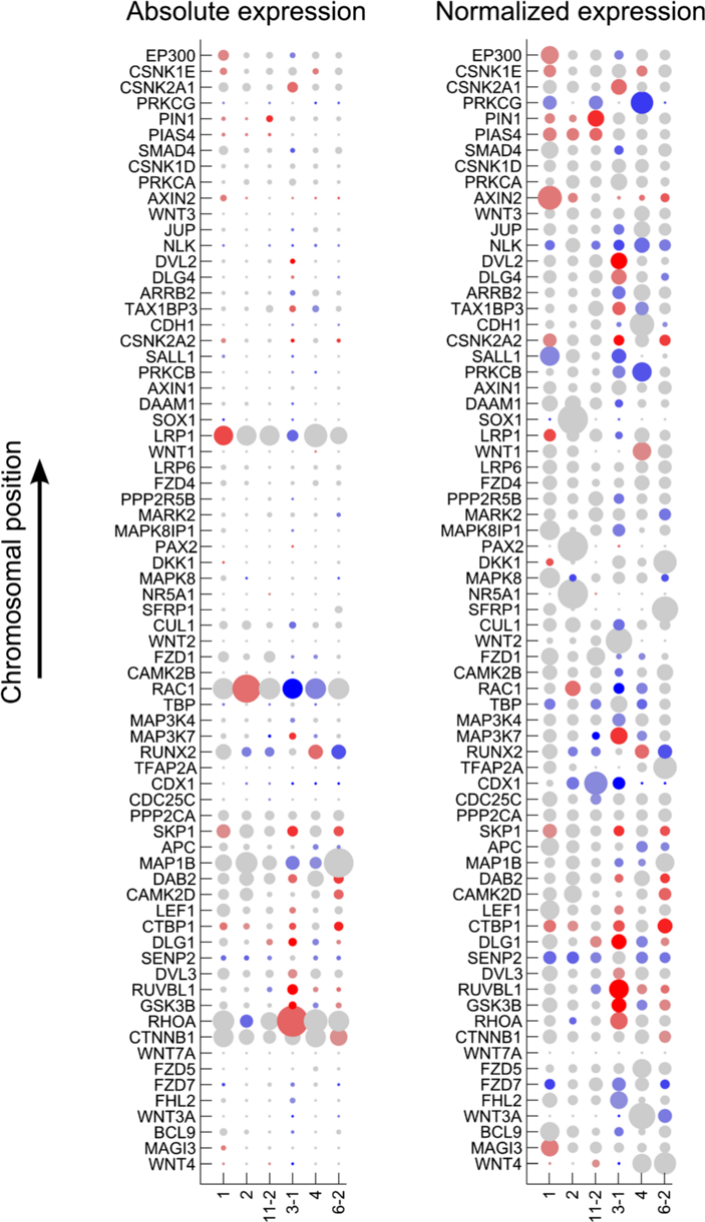
**mRNA expression of genes in the Wnt signaling pathway.** Circle color indicates copy number (red are amplified, blue are deleted). Circle size indicates mRNA expression level calculated based on RNA-seq reads. Absolute expression: the size of circles is based on the absolute read count (Left panel). Normalized expression: the size of circles is relative to the average expression (Right panel). Primary data for aCGH and RNA-seq can be found in Additional files 1 and 2.

**Figure 4:**
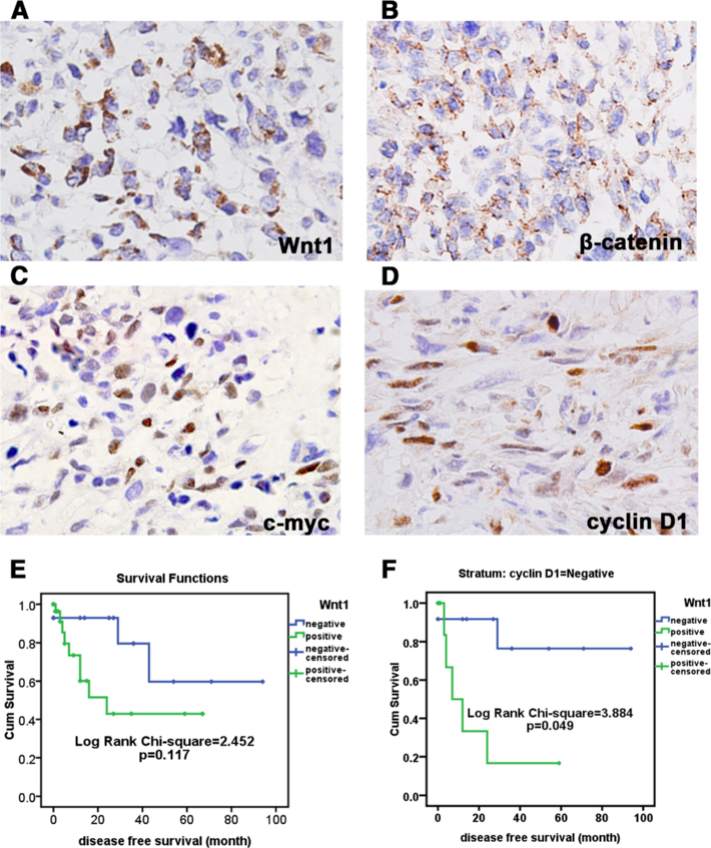
**Protein expression of the Wnt signaling pathway and its role in survival. A**. WNT1 protein expression (IHC, 10 × 40). **B**. β-catenin protein expression (IHC, 10 × 40). **C**. c-myc protein expression (IHC, 10 × 40). **D**. Cyclin D1 protein expression (IHC, 10 × 40). **E**. Disease free time of WNT1-negative patients was not significantly longer than WNT1-positive patients. **F**. K-M survival analysis showed that patients negative for both WNT1 and cyclin D1 expression had significantly longer disease free survival time.

To determine whether there was an association between gene copy number and mRNA expression of Wnt pathway genes, we compared transcriptome sequencing data of six osteosarcoma samples (Additional file 1) with aCGH analysis performed on their genomic DNA (Additional file 2). We found that the deletion of certain genes was associated with low mRNA expression, such as *NLK, SOX1, MAPK8, MAP1B,* and *FZD7* genes (Figure 3).To further explore the expression level of Wnt signaling pathways and its effect on the downstream signaling, WNT1, β-catenin, c-myc, and cyclin D1 protein expression was measured by IHC in 46 osteosarcoma samples (Figure 4A-D). WNT1 protein expression was detected predominantly in cytoplasm. β-catenin protein expression was observed in the membrane and cytoplasm but not in the nucleus. c-myc and cyclin D1 protein expression were detected in the nucleus. No protein expression was detected in negative control samples.

WNT1 protein expression was detected in 69.6% (32/46) of the osteosarcomas (Figure 4A), however no β-catenin protein expression was observed in the nucleus (Figure 4B). β-catenin protein expression was detected in the membrane and cytoplasm of 69.6% (32/46) of the osteosarcomas (Figure 4B), negative c-myc protein expression was recorded for 52.2% (24/46) of osteosarcomas and negative cyclin D1 protein expression was recorded for 47.8% (22/46) of osteosarcomas (Figure 4C-D). Compared with previously published reports of c-myc and cyclin D1 expression frequencies in other tumors, such as endometrial carcinoma [22,23], these detection frequencies (47.8% (22/46) c-myc-positive and 52.2% (24/46) cyclin D1-positive) are low. Combined with the low levels of mRNA expression for Wnt pathway genes, these data, especially the lack of β-catenin protein expression in the nucleus, suggest that the Wnt signaling pathway may be inactive.

### Negative expression of WNT1 and cyclin D1 is associated with longer disease-free survival

While the expression of WNT1, β-catenin, c-myc, and cyclin D1 had no correlation with clinicopathological factors, WNT1 expression had significant positive correlation with β-catenin (χ^2^ = 15.97, P = 0.001, Pearson’s R = 0.59), c-myc (χ^2^ = 5.62, P = 0.018, Pearson’s R = 0.35), and cyclin D1 expression (χ^2^ = 11.58, P = 0.001, Pearson’s R = 0.50). β-catenin protein expression had significant positive correlation with c-myc (χ^2^ = 5.62, p = 0.018, Pearson’s R = 0.35) and cyclin D1 (χ^2^ = 16.36, p = 5.25E-5, Pearson’s R = 0.6). Because β-catenin is a key mediating factor and c-myc/cyclin D1 are key targets regulated by the Wnt signaling pathway, these consistent relationships suggest that the initial signal, mediating factor, and downstream events of the Wnt signaling pathway may all be inactivated in human osteosarcoma.To detect the effect of the Wnt signaling pathway on survival, the disease-free and total survival of patients were analyzed. Even though single protein expression of β-catenin, c-myc and cyclin D1 showed no significant effect on disease-free survival, K-M survival analysis showed that WNT1-negative patients had a trend towards longer disease-free survival than WNT1-positive patients, although this was not statistically significant (Log Rank = 2.452, P = 0.117) (Figure 4E).Patients negative for all of WNT1, β-catenin and cyclin D1/c-myc protein expression might be considered to have an inactivated Wnt signaling pathway. Therefore, the survival of such patients will reflect the effect of inactivation of the Wnt signaling pathway on survival. Indeed, our data showed that in the patients who were negative for WNT1 protein expression, those who were also negative for cyclin D1 expression had significantly longer disease-free survival than patients with positive cyclin D1 expression (Log Rank = 3.884, P = 0.049) (Figure 4F). However, expression of none of the proteins examined had significant correlation with the overall survival of human osteosarcoma patients.

## Discussion

In this study we describe, for the first time, significant deletion of genes involved in the Wnt signaling pathway, implying genetic inactivation of this important signaling pathway, in human osteosarcoma. Supporting this, transcriptome analysis determined that mRNA expression of genes in the Wnt signaling pathway was reduced. In addition, at the protein level, nuclear β-catenin expression was not observed and c-myc/cyclin D1 protein were detected at lower frequencies compared with the frequencies observed in other tumors. Our results are in agreement with published data from Cai and colleagues, who reported that the Wnt pathway is inactivated in bone cancers [8]. Furthermore, similar results were reported by Matushansky and Gregory, indicating that inactivation of the Wnt pathway contributes to tumorigenesis in so-called malignant fibrous histiocytoma and melanoma [17,21,24]. In contrast to these results, most previous studies have suggested that active Wnt signaling contributes to osteosarcoma development [6,7]. Furthermore, it is reported that the Wnt pathway is transcriptionally active in radiation-induced rat osteosarcomas [25], and that the Wnt/β-catenin pathway antagonists, curcumin and PKF118-310, demonstrate anti-tumor activity against human osteosarcoma cells [26]. These seemingly conflicting results suggest that the complexity of this important signaling pathway is still poorly understood in human osteosarcoma [5].

An interesting but unanswered question raised in the present study is: at which time during osteosarcoma progression is the Wnt signaling pathway inactivated. As inactivation of the Wnt signaling pathway is associated with longer disease-free survival, it suggests that this inactivation may occur at an early time point in osteosarcoma progression. However, more investigations in vivo and in vitro are required before this question can be answered.

## Conclusions

Based on evidence at the genomic, our data suggest that the Wnt signaling pathway may be genetically inactivated in osteosarcoma. These results remind us of the complexity of this important signaling pathway.

## Competing interests

The authors declare that they have no competing interests.

## Authors’ contributions

XD, JY, and DY carried out the molecular genetic studies, participated in the sequence alignment and drafted the manuscript. XD, DY, and WT carried out the immunohistochemistry. JY, DY, and ZZ participated in the design of the study. JY and ZZ conceived of the study, and participated in its design and coordination and helped to draft the manuscript. All authors read and approved the final manuscript.

## Pre-publication history

The pre-publication history for this paper can be accessed here:

http://www.biomedcentral.com/1471-2407/14/450/prepub

## Supplementary Material

Additional file 1 Gene differential expression of osteosarcoma samples. Click here for file

Additional file 2 The significant deletion of Wnt signal pathway genes. Click here for file
